# Ultrasound-Guided Percutaneous Irrigation of Rotator Cuff Calcific Tendinopathy (US-PICT): Patient Experience

**DOI:** 10.1155/2020/3086395

**Published:** 2020-06-10

**Authors:** Domenico Albano, Angelo Gambino, Carmelo Messina, Vito Chianca, Salvatore Gitto, Simone Faenza, Massimo Galia, Luca Maria Sconfienza

**Affiliations:** ^1^Sezione di Scienze Radiologiche, Dipartimento di Biomedicina, Neuroscienze e Diagnostica Avanzata, Università degli Studi di Palermo, 90127 Palermo, Italy; ^2^IRCCS Istituto Ortopedico Galeazzi, 20161 Milano, Italy; ^3^Dipartimento di Scienze Biomediche per la Salute, Università degli Studi di Milano, 20100 Milano, Italy; ^4^Azienda Sociosanitaria Territoriale PINI-CTO, Milan, Italy

## Abstract

**Purpose:**

To assess patients' experience of ultrasound-guided percutaneous irrigation of rotator cuff calcific tendinopathy (US-PICT).

**Methods:**

Ninety-one patients (58 females; mean age: 50.5 ± 8.3 years) treated by US-PICT (local anesthesia, single-needle lavage, and intrabursal steroid injection) answered to a list of questions regarding their experience of the procedure before treatment, immediately after treatment, and three months later. The Borg CR10 scale was used to evaluate perceived pain, discomfort during anesthetic injection, and anxiety. The Wilcoxon, Spearman's rho, linear regression, and chi-square statistics were used.

**Results:**

81/91 patients complained mild discomfort during the injection of anesthetics (2, 1-2). Pain scores during US-PICT were very low (0, 0-1), with 70% patients having not experienced pain. After treatment, we found a significant reduction of pain (before: 8, 7-8; 3-month: 3, 1-6; *p* < .001) and anxiety (before: 5, 2-7; during treatment: 2, 1-7; *p* = 0.010), with high overall satisfaction (immediately after: 10, 9-10; 3-month: 9, 7-10) and confidence in the possibility of recovery (immediately after: 9, 8-10; 3-month: 10, 8-10), respectively. Treatments performed before US-PICT were not statistically associated with pain relief (*p* = 0.389) and clinical improvement (*p* = 0.937). We found a correlation between satisfaction immediately postprocedure and confidence in the possibility of recovery (*p* = 0.002) and between satisfaction three months after treatment and clinical improvement (*p* < 0.001) and patients' reminds about the description of the procedure (*p* = 0.005) and of the potential complications (*p* = 0.035).

**Conclusions:**

US-PICT is a mildly painful, comfortable, and well-tolerated procedure, regardless of any previous treatments. Patients' satisfaction is correlated with clinical benefit and full explanation of the procedure and its complications.

## 1. Introduction

Rotator cuff calcific tendinitis (RCCT) is a common pathologic condition affecting the rotator cuff, mainly occurring in women in their forties [1–3]. Usually, patients complain of a low-grade subacute shoulder pain increasing during the night [3]. Plain radiography and ultrasound (US) are the imaging examinations of choice [4], allowing easy detection of focal calcium depositions in the RC tendons, mostly in the supraspinatus (80%) and less frequently in the infraspinatus and subscapularis tendons (15% and 5% of all cases, respectively) [2]. Conversely, magnetic resonance imaging is not generally indicated in this setting due to the well-known limitations of this imaging technique in the evaluation of RCCT, although it is considered the pivotal imaging modality to rule out other pathologic conditions of the shoulder [5–7].

RCCT is a self-limiting condition that can be totally asymptomatic in chronic phase and not in need of treatment. However, in some cases, it can represent a painful and disabling disorder, especially when considering the acute phase [3]. Discomfort intensity influences the chosen treatment: conservative (physical therapy and oral anti-inflammatory drugs) if pain is mild or more invasive (shock waves, surgery, and imaging-guided irrigation) when symptoms are more severe. Shock wave lithotripsy was proven to be not always resolving [8], and at present, there is no standard of care for RCCT [1, 9].

Over the last years, US-guided percutaneous irrigation of calcific tendinopathy (US-PICT) has become more and more widely used [10] because of its minimal invasiveness compared to surgery and its radical impact on calcifications in comparison to shock waves, since mineralized deposits are disaggregated and removed outside the tendon [11, 12]. Furthermore, it has previously described how US-PICT facilitates prompt shoulder function recovery and pain relief [13].

It is demonstrated that even interventional or minor surgical procedures may be associated with a significant psychological burden in patients, potentially generating discomfort and anxiety [14]. Regarding US-PICT, the procedure is generally performed with 16- to 21-gauge needles under local anesthesia. We are used to explain that it is a very short, simple, and well-tolerated procedure, based on what anecdotally reported from previous patients. To our knowledge, no previous studies have focused on patients' experience of US-PICT before and immediately after the procedure, as all previous literature mostly investigated on the short- and long-term clinical outcomes of the treatment only [15].

Thus, our purpose was to assess patients' experience of US-PICT to understand whether the anxiety and pain of the patients as well as the awareness and satisfaction of the procedure itself are associated with clinical outcome after treatment.

## 2. Materials and Methods

### 2.1. Patients

Institutional review board approval was obtained for this study, and patients' consent for data collection was obtained.

We included consecutive patients with symptomatic RCCT who were specifically sent to the current institution by a pertinent orthopedic surgeon and treated by US-PICT between October 2017 and April 2018 at our institution, a tertiary orthopedic referral center.

Before treatment, the indication to treat RCCT was confirmed using US by the attending radiologist. Thus, we included in the study patients with intact and symptomatic calcification, with a clinical picture justified by RCCT according to a pertinent orthopedic evaluation. US-PICT was not performed if patient was asymptomatic, if pain was not related to RCCT, and if calcification was smaller than 5 mm, fragmented, migrated into the subacromial bursa, or was eroding the humeral cortex [1]. We acknowledge that the calcification may not remain intact after previous treatments like ESWT. Indeed, US performed prior to proceed was needed to understand whether these patients were not eligible due to a fragmented calcification. We decided to include in this study also patients already subjected to other treatments since no data are available from previous studies about the influence of prior treatments. At our institution, all patients are subjected to standard shoulder radiography prior to US-PICT, in agreement with our orthopedists. However, several X-ray scans were performed in other institutions; thus, most of them were not available for our analysis. Thus, clinical contraindications (asymptomatic, symptoms not related to RCCT) were based on orthopedic evaluation, and imaging contraindications were based on US examination which allows for a better assessment of the calcification itself as stated by the most recent European guidelines [4]. Before the procedure, the radiologist explained thoroughly to the patient the different steps of the procedure, its benefits, efficacy, and potential risks and complications (including fainting, seizures, bursitis, infection, and tendon tear), which were also well described in a written consent form signed by each patient. After the procedure, patients were also instructed on how to manage pain in the subsequent days and to start physiokinesis therapy. All patients (*n* = 112) agreed to answer to a list of questions regarding their experience with US-PICT procedure before treatment and immediately after treatment. Three months later, patients agreed to be contacted by phone to complete the last part of the list of questions, but among 112 patients, 21 were lost to follow-up. Thus, in the index period, 91 patients (58 females; mean age: 50.5 years, range: 32-74 years) were included in our analysis.

### 2.2. List of Questions

A medical doctor from our institution, not included among the study investigators, administered a list of questions to the patients. The questionnaire consisted of three different sections to be filled separately: (i) before and (ii) immediately after treatment. The third section of the list of questions was filled by one of the investigators when patients were contacted by phone at three months after treatment. The list consisted of 18 questions (*n* = 4 administered before, *n* = 9 immediately after, and *n* = 5 at three months after treatment), requiring either Borg CR10 scale score rating (*n* = 13 questions), yes/no answer (*n* = 2 questions), or open answer (*n* = 3 questions). The full list of questions is reported in Table 1. The “Borg CR10 scale” is a validated 11-point scale (ranging from 0 to 10) and a general method for measuring most kinds of perceptions and experiences, including pain and also perceived exertion. It can be used to measure not only taste and smell, loudness and noise, and brightness and other sensations but also moods and emotions (e.g., discomfort and anxiety). The scale is commonly used for measuring angina pain and breathlessness (dyspnea), musculoskeletal pain, and other kinds of somatic symptoms [16].

### 2.3. US-PICT Procedure

US-PICT was performed according to what already published in literature [11, 13, 17] by two different radiologists with 12 and 7 years of experience in this type of procedure. In brief, after US-guided injection of local anesthesia (10 ml of 2% lidocaine chlorhydrate) into the skin/subcutaneous tissue, the subacromial-subdeltoid bursa, and around the calcification, one 16-gauge needle was inserted within the calcification under continuous US monitoring. The procedure was performed with warm saline (heated to 42°C (107°F)). Calcifications were washed with a 10 mL syringe of saline (NaCl 0.9%) with repetitive pushing and releasing of the syringe plunger. This creates a reflux mechanism which allows to withdraw from patient's shoulder saline solution and the disaggregated calcium deposits. The procedure is repeated until the flushed fluid is free of visible calcium. Then, 1 mL of methylprednisolone acetate was injected inside the subacromial bursa under US guidance. After the procedure, all patients were advised to take oral painkillers for five days (1000 mg of paracetamol twice a day or even 10 mL of ketorolac tromethamine in case if needed due to strong pain unrelievable by paracetamol), to avoid arm elevation over the shoulder for one week, and then to undergo physiokinesis therapy for one month (exercises in passive mobilization, instrument assisted active mobilization, and active mobilization).

### 2.4. Statistical Analysis

Variables were summarized as median and interquartile range unless otherwise stated. We used the Wilcoxon test to compare Borg-perceived pain scores before treatment (question #1) and at three months (question #12), anxiety before (question #2) and during the procedure (question #5), and satisfaction immediately after the procedure (question #7) and at three months after treatment (question #15). The chi-square test was used to evaluate whether pain relief and improvement of clinical picture were related to other treatments performed prior to US-PICT. The Spearman's rho correlation coefficient was used to evaluate the correlation of anxiety before treatment with reminds of the patient about the description of the procedure, patient's reminds of the potential complications, and confidence in the possibility of recovery. A linear regression was used to study the relationship between satisfaction immediately after the procedure and at three months with patient's reminds about the description of the procedure/potential complications, confidence in the possibility of recovery, improvement of clinical picture, and pain relief. A *p* value lower than 0.05 was considered as statistically significant. Statistical analysis was performed using the SPSS software (v. 24, IBM, Armonk, NY).

## 3. Results

For 39/91 patients (43%), US-PICT represented the first treatment to resolve RCCT, while 23/91 (25%) patients already underwent extracorporeal shock waves without benefit, 5/91 patients (5%) received steroid injection, and 24/91 patients (26%) had both shock waves and steroid injections before US-PICT. Treatments performed before US-PICT were not statistically associated with pain relief (*p* = 0.389) and clinical improvement (*p* = 0.937). The procedure could be completed in all cases, and calcium could be obtained in all patients. All procedures were free from any immediate complications. Full patients' data is reported in Table 2.

Eleven patients reported to feel fear for treatment failure; 10, generic fear for needles; and 2, fear for anesthesia. Eighty out of 91 patients (88%) complained mild discomfort during the injection of anesthetics (2, 1-2). Median value of Borg-perceived pain score during the lavage was 0 (0-1; 0-2), with most of the patients (64/91, 70%) reporting no pain during the lavage, while the remaining patients reported a maximum Borg-perceived pain score of 2. After the procedure, we found a significant reduction of median values of pain (before: 8, 7-8; at three months: 3, 1-6; *p* < 0.001) and anxiety (before: 5, 2-7; during treatment: 2, 1-7; *p* = 0.010). There was no significant correlation between patients' anxiety and patient's reminds of the description of the procedure, patient's reminds of the potential complications, and confidence in the possibility of recovery (*r* values ranging between -0.186 and 0.001; *p* > 0.244). Results regarding pain, discomfort, and anxiety are reported in Table 3. We also found high median values of overall satisfaction (immediately after: 10, 9-10; at three months: 9, 7-10) and confidence in the possibility of recovery (immediately after: 9, 8-10; at three months: 10, 8-10), respectively. There was a significant increase of patients who would have recommended the procedure from the questionnaire provided immediately after the US-PICT and that provided at three months (*p* = 0.002).

Patients reported to be highly aware of what the procedure consisted of and its potential risks (10, 9-10), as well as the measures to be taken in the event of complications or problems (9, 8-10). Ninety-two percent of patients followed the radiologist's suggestions including medications and physical therapy after the procedure and did not require additional treatments. Among the remaining patients (8%) who did not undergo physical therapy, one required shock waves.

We found a correlation between satisfaction immediately after the procedure and confidence in the possibility of recovery (*p* = 0.002) and between satisfaction three months after US-PICT and clinical improvement (*p* < 0.001) and reminds of the patient about the description of the procedure (*p* = 0.005) and about the potential complications (*p* = 0.035). Statistical results of linear regression are reported in Table 4.

## 4. Discussion

Our main findings are that US-PICT is a mildly painful, very well-tolerated procedure, with high values of overall satisfaction and confidence in the possibility of recovery. Also, we found no association between previous treatments and pain relief at US-PICT, while correlation was found between patients' satisfaction and clinical improvement, patient's reminds about the description of the procedure, and patient's reminds of the potential complications.

US-PICT is recognized as a very minimally invasive procedure to treat RCCT, especially in respect to arthroscopy. It has the advantage of requiring a small amount of local anesthetics, short procedure time, no immobilization and hospitalization, and immediate return to work. However, the perception of this invasiveness mostly regards health operators, while patients are generally not totally aware of how the procedure works despite several video clips can be found on the Internet. For this reason, tight interaction between the physician performing the procedure and patients is crucial, to understand motivation of patients and to fully explain the procedure and its advantages and potential complications. Data of our study support this hypothesis, which is very similar to what has been demonstrated in several different clinical situations [18–21], but was never been tested before for US-PICT.

Pain during the procedure is also another crucial point. The use of small syringe needles associated with 10 mL local anesthetics seems to be sufficient to perform the procedure with minimal discomfort. This is what generally reported in literature, although no specific data on the topic have ever been reported [22–24]. Our data show that 70% of patients did not report any pain during the lavage. As expected, about 90% of patients reported mild procedural discomfort during the injection of local anesthetic, due to the sensation of burning induced by the drug, both in the subcutaneous tissues and while injecting in the bursa. Although they represent the majority of our series, the reported discomfort is overall low. This data confirms that US-PICT is really a minimally invasive and well-tolerated procedure.

Independently of emotional high acceptance, US-PICT seems to be an overall satisfying experience both immediately after treatment and after three months, so that nearly all patients would recommend it to relatives or friends in case of need. US-PICT has also confirmed to be an effective treatment for RCCT, having become strongly recommended to treat RCCT [25]. After few months, pain level was reported as low and significantly decreased compared to the pretreatment level, as previously reported [13, 26–29].

We demonstrated that clinical outcome is not related to previous different treatments on the same calcification. In clinical practice, we are generally somewhat reluctant to treat RCCT which were already treated noninvasively without success. However, in this study, we included patients who already underwent prior treatments before the US-PICT on condition that they have clinical symptoms related to RCCT and their calcification was intact at US performed before the procedure. Also, no data are available from previous studies about the influence of prior treatment as it was usually an exclusion criterion.

Some limitations should be take into account. First, patients' experience has been investigated only a short time after treatment. Although clinical efficacy of this treatment has been investigated up to 10 years [13], nothing is known about patients' experience on the long term. Further studies may be performed during longer follow-up to explore how satisfaction may change over time. Indeed, for instance, it has been demonstrated that the differences in clinical outcome between patients subjected to US-PICT and those treated by subacromial injections of corticosteroids are evident only six months after treatment [24]. Then, we do not have a control group which was treated in a different way or with patients who were not fully aware of the procedure. This was mainly done for ethical reasons, as the current approach seems to be the best possible for patients. Last, in the present series, we included only patients treated with single-needle technique. Although one-year outcome of single- and double-needle treatment have been reported to be comparable [30], we do not know whether the use of two needles may impact on patients' experience.

## 5. Conclusions

In conclusion, US-PICT is a mildly painful and well-tolerated procedure, regardless of any previous treatments. Patients' satisfaction was correlated with clinical benefit and full explanation of the procedure and its potential complications.

## Figures and Tables

**Table 1:** List of questions administered to patients before, immediately after, and at three months from treatment.

Questions before treatment	(1)	Which is your pain intensity now?	Score 0 to 10
	(2)	Which is your anxiety regarding the procedure before treatment?	Score 0 to 10
	(3)	Reason(s) of anxiety before treatment?	*Open field (optional)*
	(4)	What do you expect from this treatment?	*Open field (optional)*

Questions immediately after treatment	(5)	Which was your discomfort intensity during the injection of local anesthetics?	Score 0 to 10
	(6)	Which was your pain during the lavage?	Score 0 to 10
	(7)	Which was your anxiety during treatment?	Score 0 to 10
	(8)	Reason(s) of anxiety during treatment?	*Open field (optional)*
	(9)	Which is your overall satisfaction of the treatment?	Score 0 to 10
	(10)	Would you recommend US-PICT to others?	Score 0 to 10
	(11)	Did you receive an appropriate explanation of risks and complications of this treatment?	Score 0 to 10
	(12)	Are you aware of measures to take in case of complications?	Score 0 to 10
	(13)	Which is your confidence in overall improvement of your condition?	Score 0 to 10

Questions at three months from treatment	(14)	Which is your pain intensity now?	Score 0 to 10
	(15)	Did you comply with our suggestions after treatment?	(Yes-no)
	(16)	Would you recommend US-PICT to others?	(Yes-no)
	(17)	Which is the overall satisfaction of this treatment?	Score 0 to 10
	(18)	Did you undergo further treatments for your calcific tendinopathy?	Score 0 to 10

**Table 2:** Demographics of 91 patients undergoing US-PICT.

No. of patients	91
Gender	
Men	33 (36%)
Women	58 (64%)
Age	
Mean ± sd (range)	50.5 ± 8.3 (32-74)
Affected shoulder	
Right	60 (66%)
Left	31 (34%)
Tendon	
Supraspinatus	65 (71%)
Infraspinatus	11 (12%)
Supraspinatus-infraspinatus	5 (5%)
Subscapularis	10 (11%)
Teres minor	0
Duration of symptoms	
0-6 months	21 (23%)
7-12 months	28 (31%)
13 months or more	42 (46%)
Treatments prior to US-PICT	
Shock waves	23 (25%)
Steroid injection	5 (5%)
Both treatments	24 (26%)
None	39 (43%)

sd = standard deviation; US-PICT = ultrasound-guided percutaneous irrigation of calcific tendinopathy.

**Table 3:** Results regarding pain, discomfort, and anxiety.

No. of patients	91
Pain before the procedure	
1-3	2 (2%)
3-6	19 (21%)
7-9	60 (66%)
10	10 (11%)
Discomfort during anesthetic injection	
0	11 (12%)
1-3	81 (88%)
Pain during the procedure	
0	64 (70%)
1-3	25 (30%)
Pain at three months	
0	9 (10%)
1-3	53 (58%)
4-6	21 (23%)
7-9	8 (9%)
Anxiety before the procedure	
0	1 (1%)
1-3	35 (38%)
4-6	27 (30%)
7-9	22 (24%)
10	6 (7%)
Anxiety during the procedure	
1-3	53 (58%)
4-6	14 (15%)
7-9	20 (22%)
10	4 (4%)
Pain before the procedure vs. pain three months after the procedure	*p* < 0.001
Anxiety before the procedure vs. anxiety during the procedure	*p* = 0.010

The “Borg CR10 scale” is a validated 11-point scale (ranging from 0 to 10) and a general method for measuring most kinds of perceptions and experiences, including pain and also perceived exertion.

**Table 4:** Statistical results of the linear regression used to study the relationship between satisfaction immediately after the procedure and at three months with patient's reminds about the description of the procedure, patient's reminds of potential complications, confidence in the possibility of recovery, improvement of clinical picture, and pain relief.

		*p*
Satisfaction immediately after the procedure	Patient's reminds about the description of the procedure	0.483
	Patient's reminds of the potential complications	0.194
	Confidence in the possibility of recovery	0.002
	Improvement of clinical picture	0.493
	Pain relief	0.517

Satisfaction three months after the procedure	Patient's reminds about the description of the procedure	0.005
	Patient's reminds of the potential complications	0.035
	Confidence in the possibility of recovery	0.144
	Improvement of clinical picture	<0.001
	Pain relief	0.071

Note: a *p* value lower than 0.05 was considered as statistically significant.

## Data Availability

All data are fully available without restriction. Data are available from the internal database of IRCCS Istituto Ortopedico Galeazzi, Milano, Italy, upon Ethics Committee approval for researchers who meet the criteria for access to confidential data. The corresponding author should be contacted if someone wants to request the data.
